# Improving mental health in chronic care in general practice: study protocol for a cluster-randomised controlled trial of the Healthy Mind intervention

**DOI:** 10.1186/s13063-024-08115-8

**Published:** 2024-04-23

**Authors:** Anne Søjbjerg, Anna Mygind, Stinne Eika Rasmussen, Bo Christensen, Anette Fischer Pedersen, Helle Terkildsen Maindal, Viola Burau, Kaj Sparle Christensen

**Affiliations:** 1 Research Unit for General Practice, Bartholins Allé 2, 8000 Aarhus C, Denmark; 2 Department of Public Health, Aarhus University, Bartholins Allé 2, 8000 Aarhus C, Denmark; 3 Department of Clinical Medicine, Aarhus University, Palle Juul-Jensens Boulevard 99, 8200 Aarhus N, Denmark

**Keywords:** Diabetes mellitus, Type 2, Heart diseases, Chronic disease, Mental health, Psychosocial intervention, Problem-solving therapy, Randomised controlled trial (publication type), General practice, Primary healthcare, General practitioners, Nurses

## Abstract

**Background:**

Mental health issues are common among patients with chronic physical conditions, affecting approximately one in five patients. Poor mental health is associated with worse disease outcomes and increased mortality. Problem-solving therapy (PST) may be a suitable treatment for targeting poor mental health in these patients. This study protocol describes a randomised controlled trial of the Healthy Mind intervention, a general practice-based intervention offering PST to patients with type 2 diabetes and/or ischaemic heart disease and poor mental well-being.

**Methods:**

A stepped-wedge cluster-randomised controlled trial with 1-year follow-up will be conducted in Danish general practice. At the annual chronic care consultation, patients with type 2 diabetes and/or chronic ischaemic heart disease will be screened for poor mental well-being. Patients in the control group will be offered usual care while patients in the intervention group will be offered treatment with PST provided by general practitioners (GPs) or general practice staff, such as nurses, who will undergo a 2-day PST course before transitioning from the control to the intervention group. The primary outcome is change in depressive symptoms after 6 and 12 months. Secondary outcomes include change in mental well-being, anxiety, and diabetes distress (patients with type 2 diabetes) after 6 and 12 months as well as change in total cholesterol levels, low-density lipoprotein (LDL) levels, and blood glucose levels (patients with diabetes) after 12 months. Process outcomes include measures of implementation and mechanisms of impact. We aim to include a total of 188 patients, corresponding to approximately 14 average-sized general practices.

**Discussion:**

The Healthy Mind trial investigates the impact of PST treatment for patients with chronic disease and poor mental well-being in general practice. This will be the first randomised controlled trial determining the effect of PST treatment for patients with chronic diseases in general practice. The results of this study will provide relevant insights to aid GPs, and general practice staff manage patients with poor mental well-being.

**Trial registration:**

ClinicalTrials.gov NCT05611112. Registered on October 28, 2022.

## Administrative information

**Table:** 

Title {1}	Improving mental health in chronic care in general practice: study protocol for a cluster-randomised controlled trial of the Healthy Mind intervention
Trial registration {2a and 2b}	Clinical trial registry: NCT05611112 registered on October 28th 2022. Ethical approval: Central Denmark Region Committee on Health Research Ethics, study ID: 1–10-72–68-22
Protocol version {3}	15.12.2023 Version 1.0
Funding {4}	The Danish Heart Foundation, the Committee for Quality Improvement and Continuing Medical Education (KEU) of general practice in the Central Denmark Region, Aarhus University, the Riisfort Foundation, the Danish Research Foundation for General Practice, the Foundation for Primary Healthcare Research of the Central Denmark Region, the Committee of Multipractice Studies in General Practice, and the Foundation for Research on Mental Disorders
Author details {5a}	Anne Søjbjerg, MD^1, 2^; Anna Mygind, MScPH, PhD^1^, Stinne Eika Rasmussen, MD^1, 2^; Bo Christensen, MD, PhD^1,2^; Anette Fischer Pedersen, MScPsych, PhD^1,3^; Helle Terkildsen Maindal, MPH, PhD^2^, Viola Burau, PhD^2^; Kaj Sparle Christensen, MD, PhD^1,2^ ^1^Research Unit for General Practice, Bartholins Allé 2, 8000 Aarhus C, Denmark, ^2^Department of Public Health, Aarhus University, Bartholins Allé 2, 8000 Aarhus C, Denmark, ^3^Department of Clinical Medicine, Aarhus University, Palle Juul-Jensens Boulevard 99, 8200 Aarhus N, Denmark
Name and contact information for the trial sponsor {5b}	Department of Public Health, Aarhus University, Bartholins Allé 2, 8000 Aarhus C, Tel: + 45 87 16 79 36 Research Unit for General Practice, Aarhus, Bartholins Allé 2, 8000 Aarhus C, Tel: + 45 87 16 78 97
Role of sponsor {5c}	The trial sponsor and funders do not play a role in the design, collection, management, analysis, and interpretation of the data; drafting of the report; or decision to submit the report for publication, including authority over these activities

## Introduction

### Background and rationale {6a}

Type 2 diabetes (T2D) and chronic ischaemic heart disease (CHD) are two of the most prevalent chronic diseases affecting approximately 8% of the global population [1, 2]. Poor mental health is common in these patient groups; psychological comorbidities such as depression and anxiety affect approximately one in five patients [3–5]. In addition to this psychological burden, poor mental health is also associated with reduced self-care activities and medication adherence [6, 7], leading to worse disease outcomes and increased mortality [4, 6, 8–10]. A comprehensive approach addressing both physical and mental aspects is recommended in routine disease management of T2D and CHD, which is mainly undertaken in primary care settings like general practice [11, 12]. General practice, with a patient-centred approach and an established provider-patient relationship, is an optimal environment for early detection and treatment of mental health issues. Additionally, implementing interventions in primary care settings may increase accessibility for vulnerable patients with, e.g., low socioeconomic status and low health literacy. Consequently, interventions aiming to improve mental health in patients with T2D and CHD could ideally take place in primary care settings.

An example of such an intervention is problem-solving therapy (PST), which is an evidence-based and effective treatment for mental health issues like depression and anxiety [13, 14]. Following a relatively short training period, healthcare professionals in primary care can deliver PST [15]. Moreover, due to the tangible and comprehensible nature of PST, it is an easily accessible treatment especially for vulnerable patients. To our knowledge, PST has not previously been integrated and evaluated in chronic care management in general practice.

Building on the PST approach, we developed the Healthy Mind intervention offering PST to patients with T2D and/or CHD and concurrent poor mental well-being in general practice. In a small-scale feasibility study, the Healthy Mind intervention was found to be well-suited for the general practice setting and was positively received by both patients and healthcare providers [16]. This paper describes a stepped-wedge cluster-randomised controlled trial of the Healthy Mind intervention.

### Objectives {7}

The overall aim of the trial is to evaluate the impact of the Healthy Mind intervention using the PST approach in general practice in patients with T2D and/or CHD and poor mental well-being.

The study aims to:

1. Evaluate the effect of PST on mental health outcomes defined as depressive symptoms, symptoms of anxiety and diabetes distress (for patients with T2D) and general mental well-being
2. Evaluate the effect of PST on somatic disease outcomes, defined as cholesterol levels and blood glucose levels (for patients with diabetes)
3. Evaluate the process outcomes, including measures of implementation and mechanisms of impact

### Trial design {8}

**Fig. 1:**
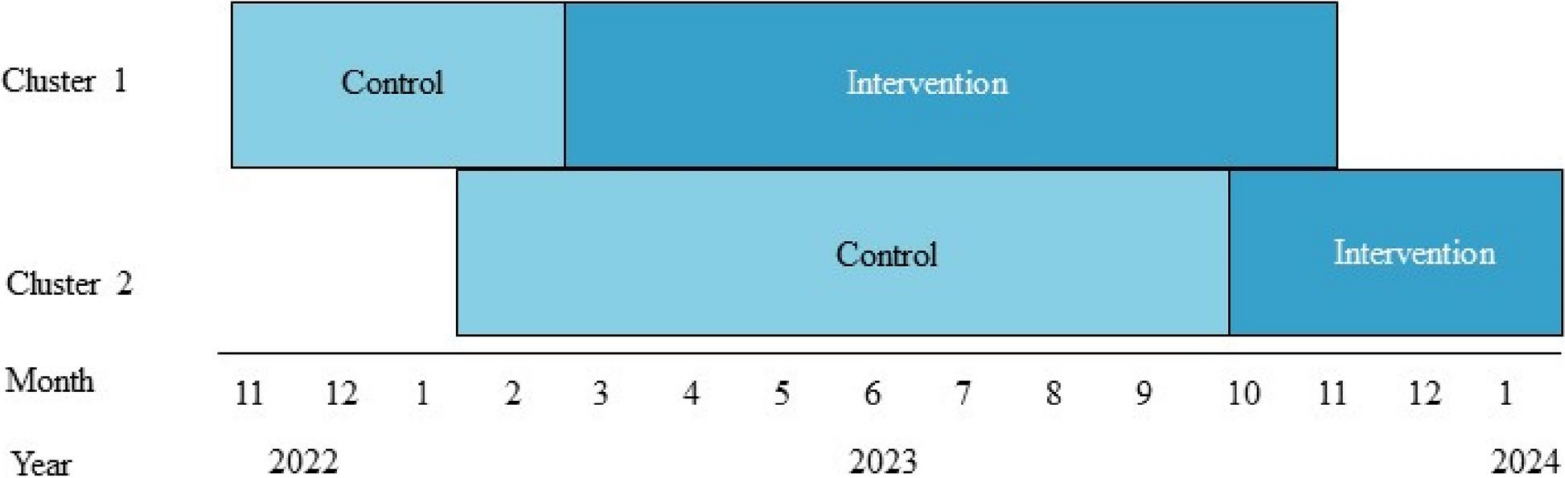
The stepped-wedge cluster-randomised trial design

The study is conducted from November 1, 2022, to January 16, 2024, with follow-up after 6 and 12 months. The design is a stepped-wedge cluster-randomised controlled trial with 1-year follow-up (Fig. 1). General practices are randomised into two clusters. Initially, all participating general practices act as the control group. After 4 months, healthcare professionals in cluster 1 undergo a 2-day PST course, following which they transition to the intervention group and begin offering the intervention to eligible patients. Healthcare providers from cluster 2 attend the PST course after 8 months and will subsequently be part of the intervention group.

## Methods: participants, interventions, and outcomes

### Study setting {9}

The study setting is Danish general practice. The Danish healthcare system is primarily tax-financed, and residents have free and equal access to general practice services. General practitioners (GPs) are self-employed, working on contracts for the public funder and offer services in their practices within the frames of a national agreement with Danish regional authorities that details which services are offered in general practice and how these are reimbursed. Nurses and other healthcare professionals (e.g. midwives, healthcare assistants) are frequently employed and trained to conduct independent consultations, including chronic care consultations in general practice [17]. In this paper, these healthcare professionals are referred to as “general practice staff”, while the term “healthcare professional” refers to both GPs and general practice staff. Currently, reimbursement is provided for up to seven 30-min talk therapy sessions per patient over a 12-month period. However, only sessions provided by GPs are remunerated [18].

Patients with T2D and CHD are typically offered annual chronic care consultations provided by either the GP or trained general practice staff. National and international guidelines on disease management encourage healthcare professionals to pay attention to psychological symptoms, but they do not offer specific recommendations on how to identify and manage mental health issues [11, 12]. Typically, annual chronic care consultations focus on specific diseases and lifestyles and less on mental health. When mental health issues are recognised, patients may be offered talk therapy, medication, or referrals to psychologists or psychiatrists.

A list of participating general practices can be provided upon request by the corresponding author.

### Eligibility criteria {10}

The following are the inclusion criteria:

1. Above 18 years of age
2. Diagnosed with T2D and/or CHD
3. Attend annual chronic care consultations
4. Poor mental well-being, defined as a WHO-5 Well-Being Index score < 50 points [19]

The following are the exclusion criteria:

1. Severe mental illness (active psychosis, suicidal mental state, or dementia)
2. Unable to understand, read, and write Danish

All general practices in the Central Denmark Region are eligible (*n* = 349). Participating healthcare professionals are GPs and general practice staff with experience in conducting independent consultations.

### Who will take informed consent? {26a}

The healthcare professional delivering the annual chronic care consultation will deliver both oral and written information about the study and obtain informed consent from eligible participants.

### Additional consent provisions for collection and use of participant data and biological specimens {26b}

No additional consent provisions are necessary and there are no ancillary studies.

### Interventions

#### Explanation for the choice of comparators {6b}

This study aims to test the effectiveness of the Healthy Mind intervention, which is provided in addition to the existing annual chronic care consultation. Therefore, usual care is the most appropriate comparator and acts as the control group.

#### Intervention description {11a}

##### Method used for intervention development

The intervention was developed using the UK Medical Research Council framework for developing complex interventions [20]. The intervention development process consisted of two phases: the initial development phase and the feasibility testing phase. Three approaches for intervention development were applied: the evidence-and-theory-based approach, the target population-centred approach, and the implementation approach [21]. During the initial phase, intervention development was informed by literature reviews, insights from a small-scale study testing PST in the general practice setting, and by attending to the existing frames in general practice [22]. In the feasibility testing phase, perspectives from healthcare providers and patients further informed the intervention development [16]. Our programme theory was refined throughout the development phase, and the intervention was adjusted accordingly.

##### Problem-solving therapy (PST)

PST is a well-established psychological treatment aiming at improving the patient’s problem-solving and coping skills [23, 24]. The assumption is that a person’s problem-solving and coping skills influence whether negative life events result in psychological symptoms, such and depression and anxiety [23, 25]. The core element of PST is training of problem-solving and coping skills by following five sequential steps: (1) listing problems and choosing one to address, (2) defining the problem in question, (3) generating solutions, (4) analysing pros and cons for each solution and choosing one, and (5) planning and undertaking the implementation of the solution. The PST provider facilitates and guides the process, while the patient takes an active role in deciding which topics to address, generating solutions, and planning and undertaking the implementation of the chosen solution. This behavioural activation is considered the pivotal component of the treatment. Patients learn to cope with their problems in a rational and systematic manner, gaining empowerment through experiential learning, realising their ability to manage encountered challenges [26].

##### The healthy mind intervention

All patients with T2D and/or CHD attending annual chronic care consultations in general practice are screened for poor mental well-being using the WHO-5 Well-Being Index (WHO-5) [19], described in detail in “Plans for assessment and collection of outcomes {18a}” section and in Table 1. In the intervention protocol, patients with a WHO-5 score < 50 points are offered PST provided by either the trained GP or practice staff. A booklet guiding patients through the five sequential steps of PST, facilitated by the healthcare professional, is provided. The booklet is designed by the research team based on the insights from the feasibility study [16]. Patients are offered up to seven 30-min PST sessions. In the control protocol, patients receive the usual care.

**Table 1:** Participant timeline

Time point	Baseline	During or immediately after the intervention	6 months	12 months
**Enrolment**				
**Eligibility screening**	X			
**Informed consent**	X			
**WHO-5 screen**^a^	X			
**Intervention**				
**Problem-solving therapy (1–7 sessions)**		X		
**Assessments**				
Primary outcome				
**PHQ-9**	X		X	X
Secondary outcomes				
**WHO-5**	X		X	X
**GAD-7**	X		X	X
**PAID-5**^b^	X		X	X
**HbA1c**^b^	X			X
**Total cholesterol**	X			X
**LDL**	X			X
Process outcomes				
Implementation				
**Fidelity**		X		
**Dose**		X		
**Reach**		X		
Mechanisms of impact				
**Mediators**		X		
**Unintended consequences**		X		

^a^Only patients with WHO-5 point score < 50 proceed in the study

^b^Only patients with T2D

##### PST training course

Before transitioning to the intervention protocol, healthcare professionals attend a 2-day PST course. The course is designed on the basis of knowledge from a previous small-scale study and further refined by insights from our feasibility study [16, 22]. Healthcare professionals are encouraged to prepare for the course by watching a 30-min video that demonstrates how PST can be delivered.

The training course comprises both a theoretical component and a component focusing on implementation. Two psychologists with expertise in PST facilitate the theoretical component, which includes lectures on PST in combination with hands-on exercises, including role play, to familiarise participants with PST and ensure that they develop sufficient skills to deliver the intervention after the course. Two members of the research team facilitate the implementation component where participants will formulate specific implementation strategies tailored to their particular general practices to ensure that the intervention is properly integrated into each participating general practice.

#### Criteria for discontinuing or modifying allocated interventions {11b}

Patients may discontinue participation at any time. The exclusion criteria ensure that patients with severe psychopathology (psychosis, suicidal mental state, or dementia) are not invited to participate in the study. If a patient develops severe psychopathological symptoms during the study, the intervention is terminated immediately, and relevant standard treatment is initiated. If a patient withdraws from the study, data collected until the withdrawal will be part of the study.

#### Strategies to improve adherence to interventions {11c}

During the PST course, healthcare providers are trained to deliver the intervention with high fidelity. The booklet used during PST sessions is designed to support adherence to the methodology of PST and facilitate patient involvement.

#### Relevant concomitant care permitted or prohibited during the trial {11d}

All usual care is allowed in both the control and the intervention group, including medical treatment of mental illness and referral to mental health specialists, e.g., a psychologist or psychiatrist.

#### Provisions for post-trial care {30}

Patients retain their usual continuous free and equal access to general practice prior to, during, and after the intervention.

### Outcomes {12}

A programme theory was created to identify anticipated mental, somatic, and process outcomes of the Healthy Mind intervention.

#### Primary outcome

The primary outcome is the mean change in depressive symptoms measured with the nine-item Patient Health Questionnaire (PHQ-9) from baseline to 6 and 12 months [27]. The PHQ-9 questionnaire is validated and frequently used in similar studies, facilitating comparison with previous studies. The PHQ-9 score was chosen as the primary outcome to maintain independence from the WHO-5 score, the measure used at patient inclusion. This decision was made to ensure the internal validity of the study.

#### Secondary outcomes

##### Mental health outcomes

Secondary outcomes include the mean change in general mental well-being measured with the WHO-5 questionnaire [19], the mean change in symptoms of anxiety measured by the seven-item General Anxiety Disorder questionnaire (GAD-7) [28], and, for patients with diabetes, the mean change in diabetes distress measured by the five-item Problem Areas In Diabetes Questionnaire (PAID-5) [29]. Outcome assessments will be made at baseline and after 6 and 12 months.

##### Somatic outcomes

Somatic outcomes include the mean change in total cholesterol levels (mmol/L), low-density lipoprotein (LDL) levels (mmol/L), and, for patients with diabetes, HbA1c (mmol/mol) from baseline to 12 months.

##### Process outcomes

Process outcomes include measures of implementation: fidelity, dose, reach, and mechanisms of impact: mediators and unintended consequences.

#### Participant timeline {13}

The participant timeline is outlined in Table 1.

#### Sample size {14}

The sample size was calculated according to the following assumptions: We expect 20% of patients with T2D and/or CHD who attend annual chronic care consultations to decline participation or be excluded from the study. We expect 15% of participating patients to report reduced mental well-being (WHO-5 score < 50 points), and 20% of patients participating in the study are expected to be lost to follow-up after 12 months. The intraclass correlation coefficient (ICC) is conservatively estimated at 0.05, reflecting a realistic expectation that a mental outcome is likely to show a larger dependence on the individual general practice than somatic outcomes, where an ICC of 0.01 is often applied. To achieve a significance level of 5% and a power of 90%, the study is required to include a total of 188 patients.

In Denmark, the size of a general practice depends on its number of practice capacities. Each general practice capacity accommodates approximately 1600 patients, with an average of 2.5 practice capacities per practice. Based on these assumptions, we need to include at least 35 practice capacities (corresponding to 14 average-size general practices) to be able to include 188 patients over a 1-year study period.

#### Recruitment {15}

##### Recruitment of general practices

An e-mail describing the study is sent by the national health authorities to all general practices in the Central Denmark Region, and interested general practices can sign up for participation. Additionally, general practices who have previously expressed an interest in participating in mental health studies and/or research projects are contacted directly by the research team.

##### Recruitment of patients

Enrolment of patients relies on the general practices to systematically recruit patients at the annual chronic care consultations. The research team will take a number of initiatives to support the workflow of patient recruitment in each general practice. Prior to commencing the study, initial online meetings will be held between two members of the research team and one GP (practice owner) from each general practice to map the staff composition, current chronic care management, and workflow. Together with the GP, the research team tailors a plan for the management of patient recruitment in each individual general practice. Immediately before the study commencement, a visit is paid to each general practice to inform healthcare professionals and administrative staff about the study and facilitate the implementation of the tailored recruitment plan. One healthcare professional from each general practice is appointed the study contact person and study champion, responsible for patient recruitment and implementation of the intervention. Subsequently, videos demonstrating how to provide oral and written information to patients will be distributed to general practices. Throughout the study period, monthly newsletters are forwarded to the participating general practices with general information about the study and individual statistics regarding patient enrolment and number of conducted PST sessions during the intervention period. Patient recruitment is monitored weekly by the research team and champions are promptly contacted by e-mail, telephone, or a visit in case recruitment rates decline, to explore the underlying reasons and support further recruitment.

### Assignment of interventions: allocation

#### Sequence generation {16a}

A computer-generated cluster randomisation is applied at the general practice level. Randomisation includes stratification by number of practice capacities and geographical location.

#### Concealment mechanism {16b}

Due to the nature of the intervention design, concealment is not possible.

#### Implementation {16c}

The allocation sequence is generated by a statistician independent of the research team. Details regarding participant and patient enrolment are outlined in the “Recruitment {15}” section.

### Assignment of interventions: blinding

#### Who will be blinded {17a}

Due to the nature and design of the intervention, blinding is not applicable. To avoid a potential impact on the healthcare providers’ professional behaviour, they only receive cursory information about PST during the control period.

#### Procedure for unblinding if needed {17b}

Blinding is not applicable in this study.

### Data collection and management

#### Plans for assessment and collection of outcomes {18a}

Information about patients is entered into the Healthy Mind website database by healthcare professionals upon enrolment. For patients with a WHO-5 score > 50 points, sex, age, and diagnosis (T2D and/or CHD) are registered. For patients with a WHO-5 score < 50 points, additional registration of social security number, name, contact information, and WHO-5 score is made.

##### Mental health outcomes

Upon enrolment, an automatically generated link to a questionnaire is sent out to participating patients with a WHO-5 score < 50 points. The questionnaire assesses the primary and secondary mental health outcomes as described above. This link is sent via email/SMS at baseline, as well as after 6 and 12 months. Reminders are sent to non-responders by email/SMS after 1 and 2 weeks, followed by a reminder telephone call after 3 weeks. Patients who are unable to access the electronic questionnaire are provided with an identical paper version at baseline and receive follow-up paper questionnaires by mail after 6 and 12 months. The questionnaires are forwarded to the research team and entered manually into the Healthy Mind database by a data manager independent of the research team. Non-responders to mailed questionnaires receive a reminder telephone call after 3 weeks. The Healthy Mind database mandates that all questionnaire items must be completed before submission, and duplicate registrations cannot be entered, ensuring comprehensive and accurate registrations. More detail on questionnaire properties is provided in Table 2.

**Table 2:** Overview of mental health outcomes in questionnaires at baseline and after 6 and 12 months

Questionnaire	Number of items	Range	Cut-off values	Measure
**PHQ-9**^a^	9	0–27	0–5: no depression 5–10: mild depression 10–15: moderate depression 15–20: moderately severe depression > 20: severe depression	Depressive symptoms
**GAD-7**^a^	7	0–21	0–4: minimal anxiety 5–9: mild anxiety 10–14: moderate anxiety > 15: severe anxiety	Anxiety symptoms
**PAID-5**^b^	5	0–20	> 7: elevated diabetes distress	Diabetes distress
**WHO-5**^c^	5	0–100	< 50: impaired mental well-being	Mental well-being

^a^4-point Likert scale (0 = not at all; 3 = nearly all of the time)

^b^5-point Likert scale (0 = not a problem; 4 = serious problem)

^c^6-point Likert scale from (0 = none of the time; 5 = all of the time)

##### Somatic outcomes

Somatic outcomes are obtained through the local LABKA II database, which contains all results of blood samples analysed in the Central Denmark Region.

##### Process outcomes

The process outcome data are part of an explanatory sequential mixed methods process evaluation, guided by the UK Medical Research Council framework for process evaluations [30, 31]. Quantitative process outcome data are collected continuously throughout the study via the Healthy Mind website database. Information about fidelity and dose is collected through questionnaires completed by healthcare providers during the registration following each PST session. Reach data are extracted from trial-monitoring records, while data on mediators and unintended consequences come from questionnaires completed by both healthcare providers and patients after the patient’s final PST session.

Qualitative data will be collected through semi-structured interviews with purposefully selected patients (*n* = 10) after their final PST session. The interview guide will focus on reach, fidelity, mediators, and unintended consequences and will be informed by quantitative process measures from each individual patient. Qualitative interview data will be transcribed verbatim and analysed thematically. The process evaluation will be supplemented by ethnographic case studies exploring the role of context.

##### Co-variates

At baseline, the Brief Health Literacy Scale for Adults (B-HLA) [32] is applied to measure the level of health literacy. Socioeconomic status data is obtained from the national registers.

#### Plans to promote participant retention and complete follow-up {18b}

General practices receive remuneration for providing information (written and oral), screening eligible patients and completing PST sessions. Remuneration will match the standard remuneration for equivalent services. This incentive will facilitate continuous patient recruitment and delivery of the intervention.

Moreover, healthcare providers are encouraged to monitor patient attendance and ensure that new PST sessions are scheduled upon cancellation or non-attendance.

The number of questionnaire items is kept at a minimum to prevent item attrition, and both electronic and paper questionnaires are available.

#### Data management {19}

In compliance with the General Data Protection Regulations (GDPR), the study is listed in the record of processing activities at Aarhus University (journal number: 2016–051-000001) [33]. Upon completion of data collection, the data set will be checked for errors, which will be resolved if possible. Computers and servers used for data management will be password-protected. The secure data management system and the Healthy Mind website database are provided by Aarhus University. Data from registers are managed through IT infrastructure provided by Statistics Denmark in accordance with the data management agreement between Statistics Denmark and Aarhus University.

#### Confidentiality {27}

Data collection, storage, and access will comply with the GDPR regulations [33]. Access to the collected data will only be granted to members of the research group.

#### Plans for collection, laboratory evaluation, and storage of biological specimens for genetic or molecular analysis in this trial/future use {33}

No collection of biological specimens for genetic or molecular analysis is made in this study.

## Statistical methods

### Statistical methods for primary and secondary outcomes {20a}

#### Primary outcome

The comparative analysis will estimate the mean change in PHQ-9 score from baseline to follow-up at 6 and 12 months. A linear mixed model will be used, adjusting for patients’ age, gender, and socioeconomic status. The cluster effect by general practice and the correlation between repeated measurements on the same patient over time will be taken into account in the multilevel analysis [34].

#### Secondary outcomes

##### Mental health outcomes

The mean change in WHO-5, GAD-7, and PAID-5 (for patients with diabetes) scores from baseline to follow-up at 6 and 12 months will be estimated using the same methods as for the primary outcome.

##### Somatic outcomes

Comparative analyses will estimate the mean change in total cholesterol levels (mmol/L), LDL levels (mmol/L) and (for patients with diabetes) HbA1c levels (mmol/mol) from baseline to 12 months using a generalised linear mixed model. The model will consider patient demographics, clustering, and time effects as described above.

##### Process outcomes

Descriptive analyses will estimate the extent of implementation (fidelity, dose, reach) and mechanisms of impact (mediators and unintended consequences). Process outcome data will be combined with effect outcome data to explore the associations between process and effect measures.

### Interim analyses {21b}

PST is a well-established treatment, which is considered safe and not expected to result in potentially serious adverse outcomes. Thus, no interim analyses are planned.

### Methods for additional analyses (e.g. subgroup analyses) {20b}

Exploratory subgroup analyses will be conducted to explore the potential differential effects of the intervention based on pre-specified characteristics of participating patients. These include sex, age, socioeconomic status, health literacy level, severity of mental health outcomes, and diagnosis (T2D or IHS). Furthermore, subgroup analysis will explore the impact of dosage received defined as a number of completed PST sessions, whether PST was provided by a GP or general practice staff, and the size of the general practice in which the intervention was delivered.

### Methods in analysis to handle protocol non-adherence and any statistical methods to handle missing data {20c}

The intention-to-treat analysis will be supplemented with a sensitivity analysis employing multiple imputation models for handling missing values to investigate the potential impact of missing data and attrition.

### Plans to give access to the full protocol, participant-level data, and statistical code {31c}

This study protocol covers all aspects of the trial. Survey data and patient outcomes are not publicly available, but deidentified data are available upon reasonable request to the corresponding author. The statistical code will be published in the manuscripts describing the effect evaluation.

### Oversight and monitoring

#### Composition of the coordinating centre and trial steering committee {5d}

Throughout the development phase, a steering committee consisting of all authors met monthly to discuss and plan the trial. During the trial, this group will meet every 3 months to discuss the trial progress. During the enrolment period, day-to-day support and continuous monitoring of the trial for operational issues will be carried out by a group of six researchers, who will meet on a weekly basis.

#### Composition of the data monitoring committee, its role, and reporting structure {21a}

The data monitoring committee comprises the day-to-day support group (six researchers) together with the data manager, who is in charge of the Healthy Mind website database (sundtsind.au.dk). The data collection is continuously monitored throughout the trial period.

#### Adverse event reporting and harms {22}

The intervention is a well-known treatment for mental health issues and is considered safe. Thus, severe adverse effects and harms are not expected. If healthcare professionals delivering the intervention suspect that the intervention has resulted in adverse events or harms, they will contact the research group immediately.

#### Frequency and plans for auditing trial conduct {23}

Formal trial auditing will not be carried out.

#### Plans for communicating important protocol amendments to relevant parties (e.g. trial participants, ethical committees) {25}

In case modifications of the protocol are found necessary, amendments will be registered on www.clinicaltrials.gov.

#### Dissemination plans {31a}

A minimum of three published articles in international peer-reviewed journals reporting on mental health outcomes after 6 months, mental and somatic outcomes after 12 months, and a process evaluation are expected. Reportings from the study will be based on the CONSORT statement extension on stepped cluster-randomised trials [35]. The study will be presented at multiple national and international conferences. Study results will be communicated to healthcare providers through professional magazines and to the public through social media and patient organisations.

## Discussion

This study will evaluate the impact of providing PST in general practice to patients with T2D and/or CHD and concurrent poor mental well-being. The chosen stepped-wedge cluster-randomised controlled trial design offers practicality and enhances data collection. Firstly, all general practices are guaranteed inclusion in the intervention arm, easing the recruitment process. Secondly, comparison between the two groups will be affected to a minimum by contextual factors, since the general practices and the healthcare professionals are identical. Thirdly, healthcare providers’ motivation to participate in the study during the control period may increase, knowing they will later transition to the intervention phase. Fourthly, cluster-level randomisation helps prevent contamination arising from treatment spillover.

Further, the embedded mixed methods process evaluation offers an in-depth understanding of how the intervention works and for whom. This comprehensive approach provides crucial insights for future implementation and valuable learning points.

There are, however, limitations in this study. Firstly, recruitment of general practices for research trials is challenging since they are self-employed often working on a tight schedule [36]. The study design and remuneration for participation are expected to facilitate recruitment. Secondly, the recruited general practices may have a particular interest in mental health, indicating that they already prioritise this in their daily practice. However, if the intervention proves effective, the effect may be more substantial in general practices that do not have the same emphasis on mental health. Thirdly, the nature of the real-life trial does not allow blinding to be applied. To reduce influence on the control group, healthcare providers are only provided with limited information about PST during the control period and participate in the PST course immediately before switching to the intervention group. Fourthly, recruitment of patients relies on screening using the WHO-5 Well-Being Index. Screening is not a part of usual chronic care in Denmark and may bring reduced mental health to the attention of patients in the control group, prompting them to seek treatment or alleviate their symptoms. Healthcare providers are instructed to provide treatment as usual for patients in the control group. Subsequent analysis of referrals and talk therapy sessions in general practice will investigate whether mental health treatment activities have increased for these patients.

The Healthy Mind intervention will be the first randomised controlled trial determining the effect of PST treatment for patients with chronic disease in general practice. Further, it will provide insights that can inform the task-shifting of psychological treatments from GPs to general practice staff. The study has broad perspectives as PST may also be applicable to other patient categories who present with mental health issues in general practice.

## Trial status

Protocol version 1.0 from December 2023. The recruitment period started in October 2022 and is scheduled to be completed in January 2024. This manuscript was submitted shortly before the inclusion termination date due to concurrent tasks faced by the authors, including reporting on findings from the preceding feasibility study. Nevertheless, the evaluation design was established well before the inclusion period began and remained unchanged after the trial commenced.

## Data Availability

Survey data and patient outcome data are not publicly available, but deidentified data are available upon reasonable request to the corresponding author.

## Abbreviations

B-HLA: Brief Health Literacy Scale for Adults
CHD: Chronic ischemic heart disease
GAD-7: General Anxiety Disorder-7
GDPR: General Data Protection Regulation
GP: General practitioner
PAID-5: Problem Areas in Diabetes-5
PHQ-9: Patient Health Questionnaire-9
PST: Problem-solving therapy
T2D: Type 2 diabetes
WHO-5: WHO-5 Mental Well-Being Index
